# The J2-Immortalized Murine Macrophage Cell Line Displays Phenotypical and Metabolic Features of Primary BMDMs in Their M1 and M2 Polarization State

**DOI:** 10.3390/cancers13215478

**Published:** 2021-10-31

**Authors:** Iolanda Spera, Ricardo Sánchez-Rodríguez, Maria Favia, Alessio Menga, Francisca C. Venegas, Roberta Angioni, Fabio Munari, Martina Lanza, Annalisa Campanella, Ciro L. Pierri, Marcella Canton, Alessandra Castegna

**Affiliations:** 1 Department of Biosciences, Biotechnologies and Biopharmaceutics, University of Bari, 70125 Bari, Italy; iolandaspera@yahoo.it (I.S.); mariafavia@hotmail.com (M.F.); martinaa.lanzaa@gmail.com (M.L.); annalisa.campanella@uniba.it (A.C.); ciro.pierri@uniba.it (C.L.P.); 2 Department of Biomedical Sciences, University of Padova, 35131 Padova, Italy; ricardo.sanchezrodriguez@unipd.it (R.S.-R.); francisca-venegas@phd.unipd.it (F.C.V.); fabio.munari@unipd.it (F.M.); 3 Fondazione Istituto di Ricerca Pediatrica Città della Speranza—IRP, 35129 Padova, Italy; roberta.angioni@unipd.it; 4 Department of Molecular Biotechnology and Health Sciences, Molecular Biotechnology Center, University of Torino, 10126 Turin, Italy; alessio.menga@unito.it

**Keywords:** macrophage, immortalized macrophages, metabolism, inflammation, immune model, cell line

## Abstract

**Simple Summary:**

Evidence of the role of macrophages in promoting cancer progression has prompted scientists to investigate innate immune cell function in order to identify targetable checkpoint for reverting the protumoral functions of macrophages. Primary cultures isolated from mice necessary to investigate the mechanisms mediating immune cell activation require expensive and time-consuming breeding and housing of mice strains. We obtained an in-house generated immortalized macrophage cell line from BMDMs. In the present study, we characterize this cell line both from a functional and metabolic point of view, comparing the different parameters to those obtained from the primary counterpart. Our results indicate that classically and alternatively immortalized macrophages display similar phenotypical, metabolic and functional features to primary cells polarized in the same way, validating their use for in vitro studies relevant to the understanding and targeting of immune cell functions within tumors.

**Abstract:**

Macrophages are immune cells that are important for the development of the defensive front line of the innate immune system. Following signal recognition, macrophages undergo activation toward specific functional states, consisting not only in the acquisition of specific features but also of peculiar metabolic programs associated with each function. For these reasons, macrophages are often isolated from mice to perform cellular assays to study the mechanisms mediating immune cell activation. This requires expensive and time-consuming breeding and housing of mice strains. To overcome this issue, we analyzed an in-house J2-generated immortalized macrophage cell line from BMDMs, both from a functional and metabolic point of view. By assaying the intracellular and extracellular metabolism coupled with the phenotypic features of immortalized versus primary BMDMs, we concluded that classically and alternatively immortalized macrophages display similar phenotypical, metabolic and functional features compared to primary cells polarized in the same way. Our study validates the use of this immortalized cell line as a suitable model with which to evaluate in vitro how perturbations can influence the phenotypical and functional features of murine macrophages.

## 1. Introduction

The role played by macrophages in different diseases, ranging from cancer to autoimmune disease, diabetes and obesity, is now established. Macrophages are cells of the immune system that are involved in the acquisition of different activation states in response to specific stimuli. Quiescent macrophages (MФ) can be activated by interferon-γ (IFNγ) and Toll-like receptor (TLR) agonists toward an inflammatory (M1-like) phenotype, thus displaying pro-inflammatory features with microbicidal and tumoricidal properties. Conversely, under interleukin-4 (IL-4), IL-13, and IL-10 (M2-like activation), macrophages release anti-inflammatory factors (IL-10, transforming growth factor b [TGFβ], and IL-1 receptor antagonist [IL-1Ra]), promoting immune suppression, debris scavenging, angiogenesis, tissue remodeling, and repair [1,2]. These polarization states associate with a distinct regulation of cellular metabolism, with LPS-activated M1 macrophages undergoing a metabolic switch to enhanced glycolysis with suppression of the respiratory chain [3,4,5]. On the other hand, IL-4 primes macrophages toward enhanced fatty acid oxidation (FAO) and mitochondrial oxidative phosphorylation (OXPHOS) [6,7,8]. This different metabolic status is not simply associated with the polarization state but is necessary to achieve polarization and the associated inflammatory and regulatory functions. Indeed, blocking FAO impairs M2 polarization, whereas inhibiting glycolysis hampers M1 polarization [9]. For these reasons, the acquisition of a polarization state in macrophages is often confirmed by assessing their metabolism. Investigating the role of macrophages in a disease context is now emerging as a fundamental step through which to understand the complex cellular mechanisms underlying pathogenesis. In cancer, macrophages play a pivotal role in regulating the tumor microenvironment, and, consequently, in promoting or suppressing the development of the disease, influencing the main features of malignant tumors, such as angiogenesis, invasiveness, metastasis and therapeutic resistance [10]. Hence, studies of the mechanisms of innate immune activation often require the availability of genetically modified mice, not only for in vivo but also for in vitro studies. The fact that macrophages are generated from these mouse strains requires a high investment in terms of costs and spaces for animal housing and breeding.

Recently, accumulating evidence has highlighted the interesting possibility that immortalization of macrophage populations could be a strategy to overcome this issue. This can be achieved with specific mouse strains using the J2 retroviral infection method of immortalization, which has been successfully applied on fetal liver macrophages, splenic macrophages, microglial cells, and bone marrow-derived macrophages (BMDM) [11,12,13,14]. This approach might allow the generation of cell lines of immortalized murine macrophages bearing the typical functional features of macrophages and, therefore, the phenotypical mimicking of their primary counterparts [11]. Furthermore, genetically modified immortalized macrophages could also be successfully produced, facilitating the investigation of relevant mechanisms of immune metabolism.

We obtained an in-house immortalized macrophage cell line with the aim of investigating the metabolic mechanisms underlying macrophage phenotypes. Taking advantage of commercial AMJ2-C11 alveolar macrophages (ATCC) as a resource of J2 retrovirus that carries the v-*raf* and v-*myc* oncogenes, BMDMs were infected and quickly immortalized [15,16,17]. In the present work, we compare the immortalized cell line with BMDMs, polarized toward an M1- and M2-like state, by evaluating the expression level of specific M1 and M2 genes and markers of inflammasome activation and by characterizing their metabolic and bioenergetic status. Our results indicate that immortalized macrophages mimic the main phenotypical and metabolic features of BMDMs, suggesting the potential utilization of this cell line as a tool to investigate specific mechanisms of macrophage polarization and identify novel ways to rewire macrophage function in cancer research. 

## 2. Material and Methods

### 2.1. J2 Retrovirus Isolation

The J2 retrovirus was obtained through the culturing of AMJ2-C11 cells (ATCC, Manassas, VA, USA), as previously reported [17]. Briefly, the AMJ2-C11 cells were cultured at 70% of confluence. After 3 days, the J2 enriched-culture medium was recovered and clarified by centrifugation at 500 g for 5 min and filtered by 0.45 µm. 

### 2.2. Primary Cell Culture and Immortalization of BMDM

The BMDMs were obtained under an institutional review board-approved protocol (University of Padova, Prot. 890/2018). Bone marrow cells were flushed from male C57BL/6 mouse femurs from 8 to 12 weeks of age, and differentiated to BMDM in RPMI medium 1640 supplemented with 10% FBS (Merck Millipore, Burlington, MA, USA) with 40 ng/mL of M-CSF (Miltenyi Biotec, Bergisch Gladbach, Germany) for 7 days. At day seven, the BMDM culture medium was removed and the cells were exposed to the J2 retrovirus enrichment medium in the presence of 3 µg/mL of Polybrene (SantaCruz Biotechnology, Dallas, TX, USA) for 2 h at 37 °C and 5% CO_2_. The RPMI medium was then added 1:1 supplemented with 10 ng/mL of MCSF and the cells were incubated for 24 h at 37 °C and 5% CO_2_; the following day, a second exposure to J2 retrovirus was performed and the cells were maintained in culture for 1 week, then the MCSF was reduced by 50% and cultured for one week more. Finally, the immortalized cells (iµΦ) were grown in the absence of cytokine and then characterized for macrophage markers. The experiments were performed at BSL2 biosafety level and according to the Good Laboratory Practice procedures implemented at the University of Padova.

The BMDM and iµΦ were polarized to M1 state with LPS 500 ng/mL (Sigma Aldrich, Saint Louis, MO, USA) and IFNγ 25 ng/mL (PreproTech, East Windsor, NJ, USA) or to M2 state with IL-4 25 ng/mL (Miltenyi Biotec).

### 2.3. Sea Horse Experiments

The metabolic parameters were calculated by using the Seahorse XF96 (Agilent, Santa Clara, CA, USA) as described previously [18]. Briefly, the polarized BMDMs were seeded in a 96-well Seahorse XF24 cell culture microplate in complete medium (10^5^ cells per well). To evaluate the Oxygen Consumption Rate (OCR), one hour before the experiment, the medium was replaced with Seahorse medium (Dulbecco’s Modified Eagle Medium (DMEM) Sigma-Aldrich) supplemented with 33 mM NaCl, 5 mM glucose, 1 mM sodium pyruvate, 15 mg/l phenol red, 2 mM glutamine, pH 7.4).

The OCR evaluation was performed by sequentially injecting the following electron transport chain inhibitors: Oligomycin A (1.5 µM), Carbonyl cyanide-4-(trifluoromethoxy)phenylhydrazone (FCCP) (1.6 µM), Antimycin A (2.5 µM) and Rotenone (1.25 µM). The parameters of mitochondrial respiration were analyzed using Wave software and the Cell Mito Stress assay report generator (Agilent). The OXPHOS parameters were calculated as follows: non-mitochondrial respiration = average after Rot/AA; basal respiration = average OCR before Oligo−average OCR after Rot/AA; ATP production = average OCR before Oligo−average OCR after Oligo; Maximal respiration = average OCR after FCCP−average after Rot/AA.

### 2.4. Real-Time PCR

The total RNA was extracted by Trizol (Invitrogen, Waltham, MA, USA) following the manufacturing protocol. The cDNA reactions were prepared with 500 ng of the total RNA using a High Capacity cDNA Reverse Transcription Kit (Applied Biosystems Thermo Fisher Scientific, Waltham, MA, USA) and a 1/10 dilution was used for the quantitative PCR. The reactions were carried out using the specific primers indicated in Table 1 and SYBR Green in the Quantum Real Time PCR system (Applied Biosystem). The data were normalized against the *Rplp0* gene expression using the comparative ΔΔCt method compared to MФ BMDMs. 

### 2.5. Western Blotting

The total protein extract was obtained with a FASP buffer. 20 μg of the total protein were separated by 4–12% Bold NuPage (Thermo Fisher Scientific) and transferred onto PVDF membranes (BioRad, Hercules, CA, USA). After blocking with albumin (Sigma-Aldrich) and incubating with primary antibodies (1:1000) of nitric oxide synthase (NOS2, Cell Signaling, Danvers, MA, USA), arginase 1 (ARG1, SantaCruz Biotechnology) or Actin-HRP (Abcam, Cambridge, UK), the membranes were incubated with an anti-rabbit peroxidase-conjugated secondary antibody (GE healthcare, Chicago, IL, USA). Chemiluminescence was obtained by using the ECL Substrate (GE healthcare), and the images were obtained with an ImageQuant LAS 500 (GE Healthcare).

### 2.6. Flow Cytometry Analysis

**Figure A1:**
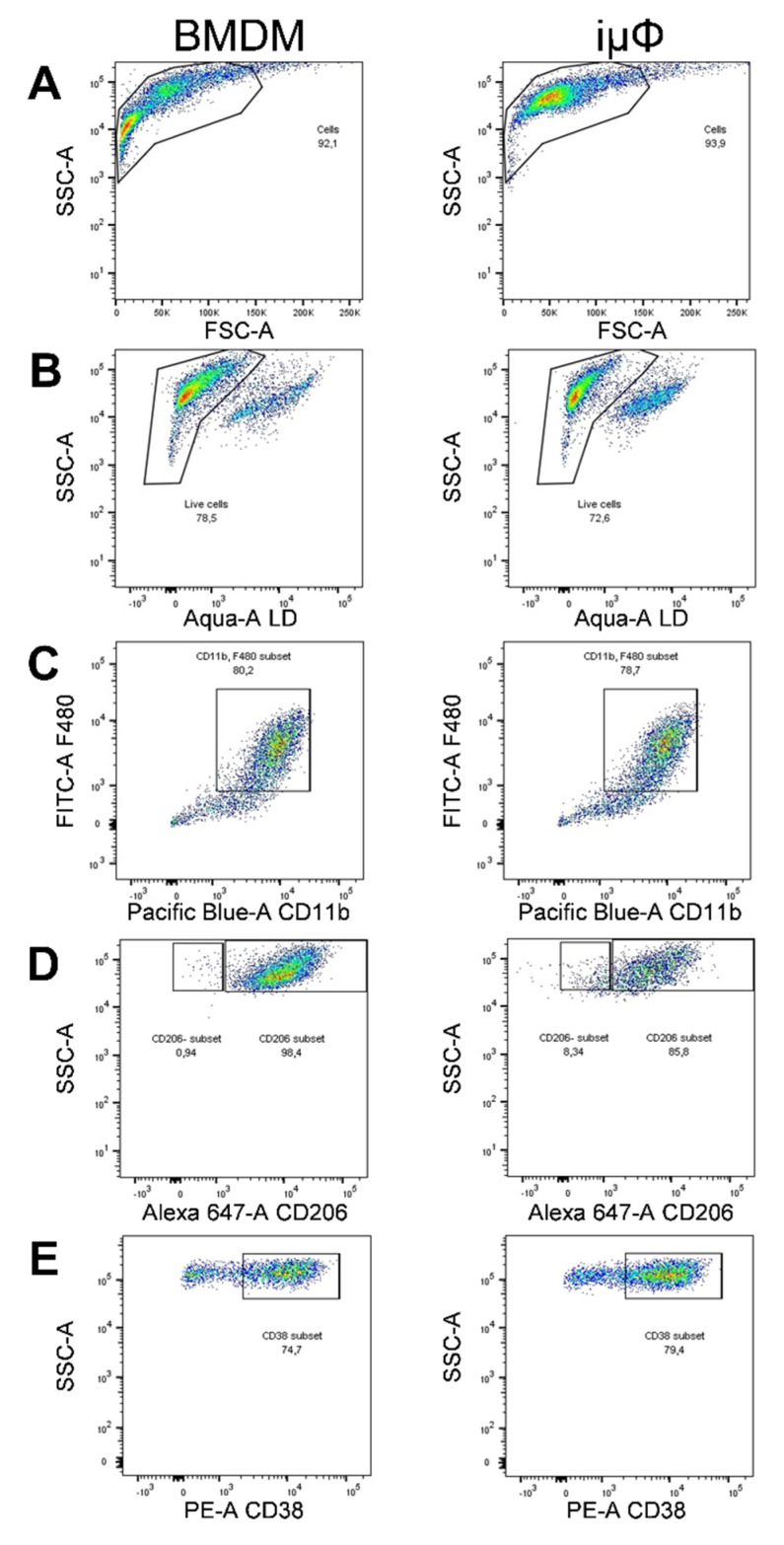
Gating strategy for FACS macrophage characterization. (**A**) BMDM and immortalized macrophages (iµФ) in their naïve form (MΦ) were selected according to morphology and granulocity (SSC-A vs. FSC-A) and data were plotted using FlowJo software. Inside of the plot, live cells were discriminated by Aqua staining (**B**) Live macrophages were identified as double-positive CD11b and F4/80 events (**C**) BMDM and iµФ were polarized with IL4 (40 ng/mL) to M2 or with LPS (500 ng/mL) and IFNγ (25 ng/mL) to M1 and the surface markers CD206 (M2, (**D**)) and CD38 (M1, (**E**)) were plotted.

The cells were collected with PBS-EDTA 2 mM and stained for flow cytometric analysis with the following antibodies for 20 min at 4 °C, after Fc-receptor blocking: anti-CD11b-PB (clone M1/70, Biolegend, San Diego, CA, USA), anti-F4/80-FITC (clone CI:A3-1, Biorad), anti-CD206-Alexa647 (clone C068C2, Biolegend) or anti-CD38-PE (clone REA616, Miltenyi Biotec). Dead cells were excluded using Live/Dead^®^dye (Invitrogen). The samples were acquired with a CitoFLEX Flow Cytometer (Beckman Coulter, Brea, CA, USA) and analyzed with FlowJo software (Tree Star, Inc., East Rutherford, NJ, USA). The gating strategy is shown in Figure A1.

### 2.7. Metabolite Quantification

For the mass spectrometry analysis, the cells were scraped in 80% methanol to extract polar metabolites. Subsequently, the samples were centrifuged and dried using a vacuum concentrator [19,20]. The dried metabolite samples were stored at −80 °C. The measurements of the metabolites were obtained with an Acquity UPLC system interfaced with a Quattro Premier mass spectrometer (Waters, Milford, MA, USA) [19,20,21,22]. The calibration curves were established using standards and processed under the same conditions as the samples, at five concentrations. The best fit was determined using a regression analysis of the peak analyte area. The multiple reaction monitoring transitions selected in the negative ion mode were *m/z* 190.95 > 110.89 for citrate, *m/z* 116.88 > 73.20 for succinate, *m/z* 132.95 > 115.20 for malate and *m/z* 144.91 > 100.97 for 2-oxoglutarate (2-OG); in the positive ion mode, the transitions selected were *m/z* 90.00 > 44.24 for alanine (Ala), *m/z* 175.20 > 70.01 for arginine (Arg), *m/z* 176.00 > 159.00 for citrulline (Cit) and *m/z* 133.00 > 70.01 for ornithine (Orn). Chromatographic resolution was achieved using HSS T3 (2.1 × 100 mm, 1.8 μm particle size, Waters) for the TCA and BEH C18 (2.1 × 50 mm, 1.7 μm particle size, Waters) for the amino acids. For all the columns, the flow rate was 0.3 mL/min.

### 2.8. ELISA

To evaluate the IL-1β release after inflammasome activation, the BMDMs and iµΦ were primed for 4 h with 100 ng/mL LPS and, for the last 15 minutes, activated with 5 mM ATP (Sigma-Aldrich). The collected supernatants were maintained at −80 °C until analysis. Their quantification was performed by ELISA (R&D, Minneapolis, MN, USA), according to the manufacturer’s instructions.

### 2.9. Macrophage-Myofibroblast Transition Assay

The macrophage to myofibroblast transition was evaluated as described previously [23,24]. Briefly, iµΦ were stimulated with 5 ng/mL TGFβ (R&D) for 5 days. Next, the co-expression of macrophage and myofibroblast markers anti-F4/80-FITC and anti-α smooth muscle actin (αSMA)-APC (Clone E184, Abcam) were measured by flow cytometry. Untreated iµΦ were used as a control.

### 2.10. Statistical Analysis

The data are shown as means ± SEM. At least three independent experiments were performed. The statistical significance of the comparisons between the groups was determined using ANOVA test, followed by post hoc Tukey test. The data were considered significant for * *p* < 0.05, ** *p* < 0.01, *** *p* < 0.001, **** *p* < 0.0001.

## 3. Results

**Figure A2:**
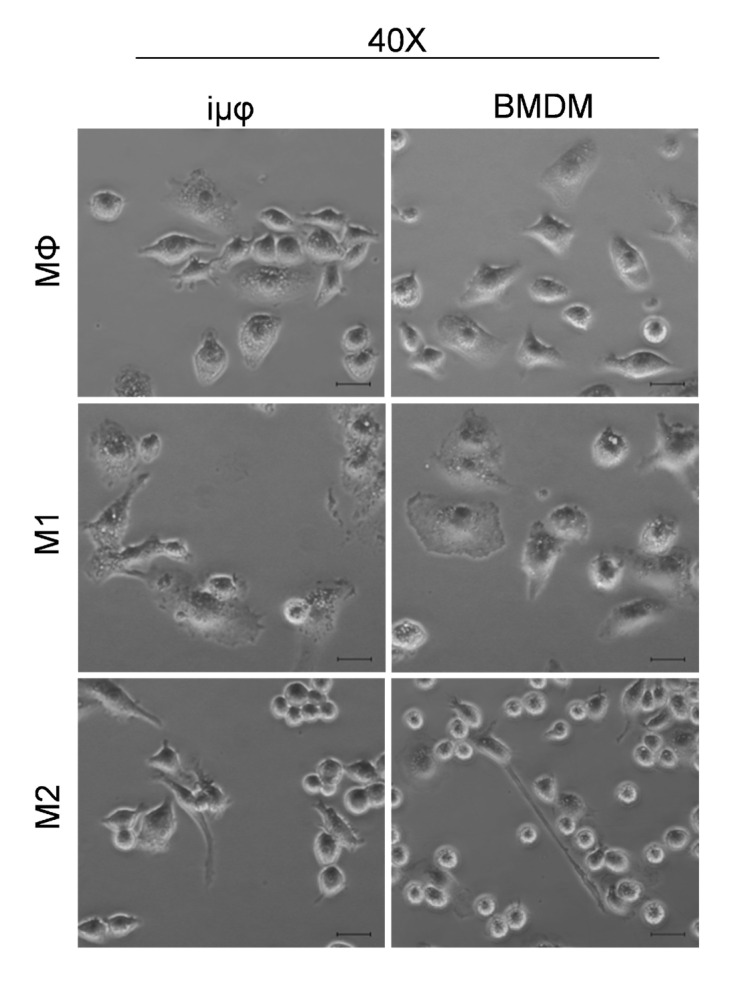
Transmission microscopy images of resting (MΦ), M1 and M2 stimulated iμΦ or BMDM macrophages. Scale bar, 10 µm.

In order to compare the two different cell types, the BMDMs and immortalized BMDMs (iμΦ) were stimulated according to the classical and alternative stimulating procedures, leading to M1-classically activated and M2-alternatively activated macrophages. The morphological similarity between the iμΦ and BMDM macrophages was visualized by light microscopy (Figure A2).

**Figure 1:**
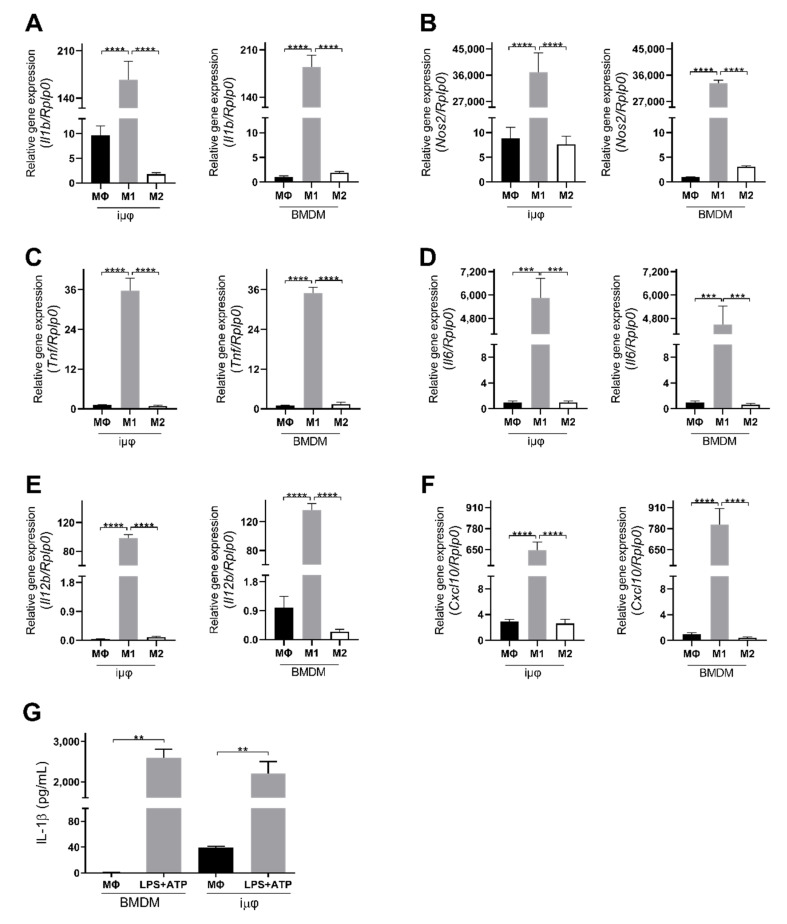
Classically (M1)-stimulated immortalized murine macrophages (iμФ) and BMDMs display similar phenotypical features. (**A**–**F**) qRT-PCR quantification of *Il1b* (**A**), *Nos2* (**B**), *Tnf* (**C**), *Il6* (**D**), *Il12b* (**E**), *Cxcl10* (**F**), specific genes in resting (MΦ), M1- and M2-stimulated iμΦ macrophages (*n* = 3) and in BMDM macrophages (*n* = 3). (**G**) Levels of IL-1β release from LPS + ATP-stimulated iμΦ and BMDM macrophages. Data are means ± SEM. ** *p* < 0.01, *** *p* < 0.001, **** *p* < 0.0001.

**Figure 2:**
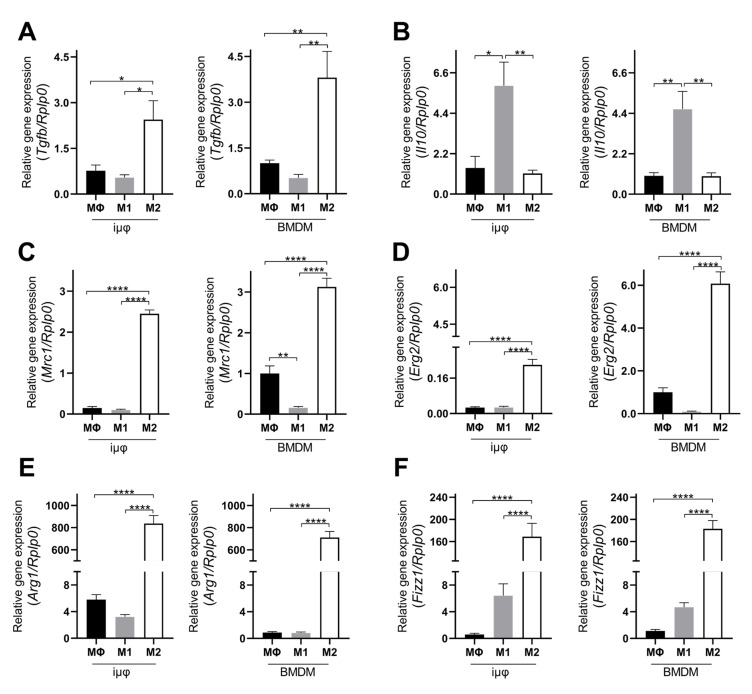
Alternatively (M2) stimulated immortalized murine macrophages (iμФ) and BMDMs display similar phenotypical features. qRT-PCR quantification of *Tgfb* (**A**), *Il10* (**B**), *Mrc1* (**C**), *Erg2* (**D**), *Arg1* (**E**) and *Fizz1* (**F**), specific genes in resting (MΦ), M1- and M2-stimulated iμΦ macrophages (*n* = 3) and in BMDM macrophages (*n* = 3). Data are means ± SEM. * *p* < 0.05, ** *p* < 0.01, **** *p* < 0.0001.

**Figure 3:**
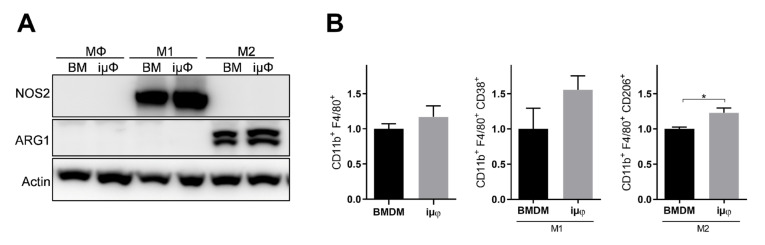
NOS2 and ARG1 protein levels and CD11b^+^ cell population are similar in M1 and M2 stimulated iμΦ and BMDM macrophages. (**A**) Representative blot images of NOS2 and ARG1 proteins expression in resting (MΦ)-, M1- and M2-stimulated iμΦ or BMDM macrophages (*n* = 3). (**B**) Flow cytometry cell analysis of CD11b^+^CD38^+^ cell population in M1-stimulated iμΦ and BMDM macrophages and for CD11b^+^CD206^+^ cell population in M2-stimulated iμΦ and BMDM macrophages. Data are means ± SEM. * *p* < 0.05. Original blot images were presented in Figure S1.

The acquisition of the different phenotypes was tested by a qPCR evaluation of specific M1 (*Il1b, Nos2*, *TNF*, *Il6*, *Il12b* and *Cxcl10*) and M2 (*Tgfb, Il10, Mrc1, Erg2, Arg1* and *Fizz1*) genes. As indicated in Figure 1, the expression levels of the M1 genes increased specifically and to a similar extent in the M1-stimulated iμΦ and BMDMs. Furthermore, as shown in Figure 2, the expression levels of the M2 genes were specifically increased in the M2-stimulated macrophages from the iμΦ and BMDMs, except for *Erg2*, which displayed a modest increase in M2-stimulated iμΦ compared to the similarly stimulated BMDMs. In support of the phenotypic features of the stimulated BMDMs and iμΦ, the NOS2 and ARG1 protein levels significantly and similarly increased in both cell types differentiated toward the M1 and M2 phenotypes, respectively (Figure 3A). These results were confirmed by using multiparametric flow cytometry, with a similar percentage of CD11b^+^CD38^+^ cell populations in the M1-stimulated iμΦ and BMDMs, and a percentage of CD11b^+^CD206^+^ cell populations in the M2-stimulated iμΦ only slightly increased compared to the BMDMs (Figure 3B). All these data clearly indicate that iμΦ respond as BMDMs to M1 and M2 stimulation and display the typical features of the M1 and M2 states, respectively. 

Furthermore, we tested the functional response of both cell types to pro-inflammatory stimuli by evaluating the inflammasome activation. Inflammasome is a functional complex assembled in response to infection or tissue injury, which senses signals from pathogen-associated molecular patterns (PAMPs) or danger-associated molecular patterns (DAMPs) to generate active caspase-1, which converts IL-1β into its mature active form. IL-1β release is then an indicator of NLRP3 inflammasome’s correct assembly. As indicated in Figure 1G, iμΦ stimulated with LPS + ATP release IL-1β to a similar extent to BMDMs stimulated in the same way, indicating that the process of the immortalization of BMDMs does not hamper the assembly of the multiprotein, active inflammasome complex.

**Figure 4:**
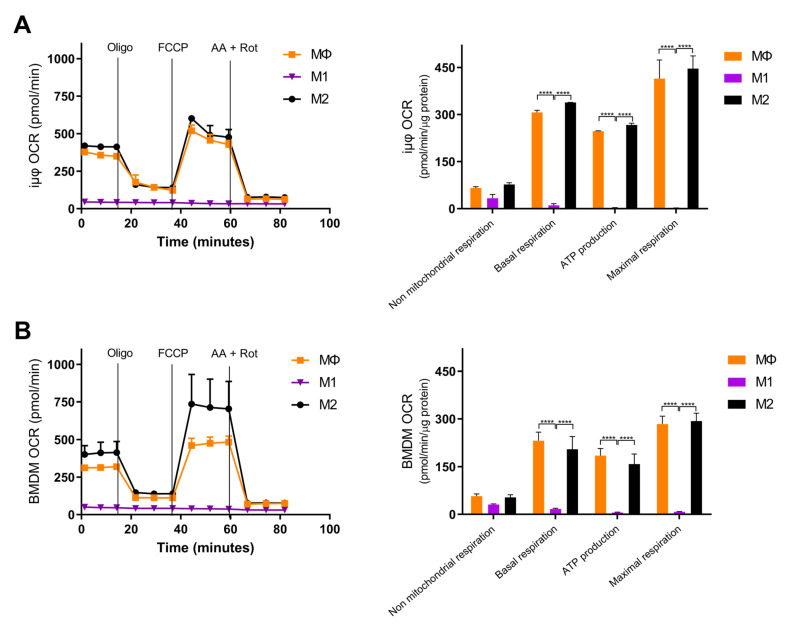
Bioenergetics are similar in M1- and M2-stimulated iμΦ and BMDM macrophages. iμΦ (*n* = 3) (**A**) and BMDMs (*n* = 3) (**B**) were seeded in Seahorse plates and stimulated for 24 h to yield resting (MΦ)-, M1- and M2-polarized macrophages. During extracellular flux analysis, cells were sequentially treated with oligomycin (1.5 µM), FCCP (1.6 µM), and antimycin A plus rotenone (AA + ROT, 2.5 µM and 1.5 µM, respectively) to assess the OXPHOS parameters from the Oxygen Consumption Rate (OCR) levels (right panels, see Materials and Methods for details). FCCP uncouples mitochondrial respiration and the corresponding OCR measurements yield data about the maximal respiratory capacity. Rotenone and antimycin A block mitochondrial complex I and III, and the residual OCR represents non-mitochondrial oxygen consumption. Data are means ± SEM. **** *p* < 0.0001.

Next, we compared the metabolic features of the M1- and M2-stimulated iμΦ with the BMDMs. Usually, polarized macrophages display distinct metabolic features, depending on the stimulus. M1 macrophages undergo a metabolic switch toward enhanced glycolysis [3,4,5], whereas mitochondrial oxidative phosphorylation (OXPHOS) is a typical feature of M2 macrophages [6,7,8]. To characterize the metabolic features of both cell types, we evaluated the mitochondrial bioenergetics in resting (MΦ)-, M1- and M2-stimulated iμΦ and compared them to the same parameters obtained with similarly stimulated BMDMs. As shown in Figure 4A,B, the OCR curve of the M2-stimulated iμΦ, which evaluates total and basal mitochondrial respiration, ATP production, proton leak, maximal respiration and spare respiratory capacity, perfectly mimicked that obtained from the M2-stimulated BMDMs, indicating, as expected, the enhanced OXPHOS and ATP production associated with M2 stimulation. The M1-stimulated iμΦ did not display a relevant OCR, similarly to the M1-stimulated BMDMs (Figure 4A,B), which was in line with the notion that under M1 stimulus, macrophages obtain ATP mainly from glycolysis [18].

**Figure 5:**
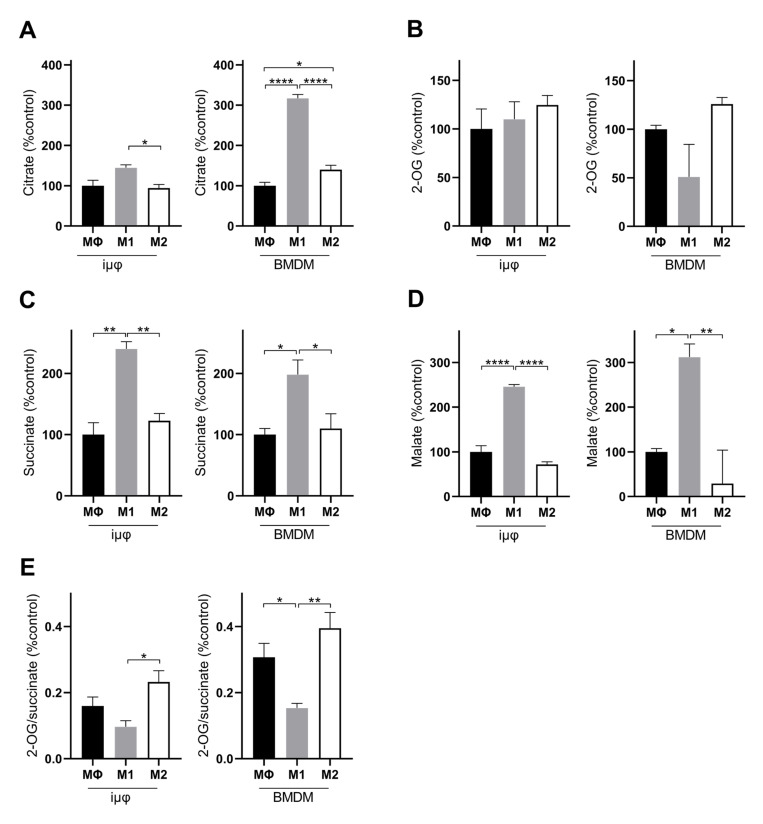
M1- and M2-stimulated iμΦ and BMDM macrophages display similar features for TCA metabolites. LC-MS/MS quantification of citrate (**A**), 2-OG, (**B**), succinate (**C**), malate (**D**) and 2-OG/succinate ratio (**E**) in MΦ-, M1- and M2- stimulated iμΦ (*n* = 3) or BMDM cells (*n* = 3). Data are means ± SEM. * *p* < 0.05, ** *p* < 0.01, **** *p* < 0.0001.

A comparison of the % changes of the intracellular key metabolites following M1 and M2 stimulation in both cell types further confirms the similarities between stimulated iμΦ and BMDMs. Although the metabolite % increase levels were different in some cases, both cell types displayed the typical metabolic features of M1 and M2 macrophages. In the M1-stimulated iμΦ and BMDMs, TCA cycle intermediates, such as citrate (Figure 5A), succinate (Figure 5C) and malate (Figure 5D), accumulated, which is a typical feature of pro-inflammatory macrophages as a result of TCA cycle interruption and OXPHOS suppression [25,26]. The citrate accumulation was higher in the M1-stimulated BMDMs than in the M1-stimulated iμΦ (Figure 5A). By contrast, no accumulation of the TCA cycle intermediates occurred in the M2-stimulated iμΦ or BMDMs (Figure 5A–D), in line with the enhanced TCA cycle flux coupled with OXPHOS typical of an M2 state [25,27]. This feature was also confirmed by the 2-OG/succinate ratio, which represents a metabolic indicator of the acquired M1 (lower ratio) versus M2 state (higher ratio) [27]. As expected, this ratio was lower in the M1, but higher in the M2-stimulated iμΦ and, similarly, in the BMDMs stimulated in the same way (Figure 5E). 

**Figure 6:**
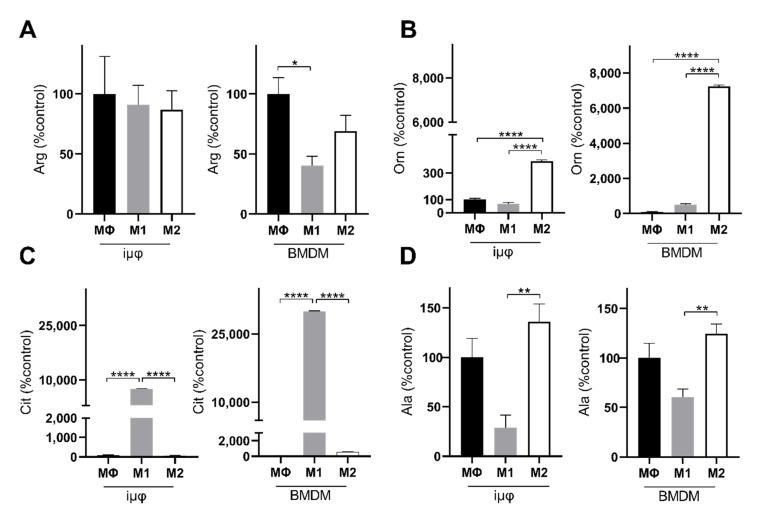
M1- and M2-stimulated iμΦ and BMDM macrophages display similar metabolic features for key amino acids. LC-MS/MS quantification of arginine (Arg, (**A**)), ornithine (Orn, (**B**)), citrulline (Cit, (**C**)) and alanine (Ala, (**D**)) in resting (MΦ)-, M1- and M2-stimulated iμΦ (*n* = 3) and BMDM macrophages (*n* = 3). Data are means ± SEM. * *p* < 0.05, ** *p* < 0.01, **** *p* < 0.0001.

The levels of alanine and arginine confirm the metabolic similarities between the M1- and M2-stimulated iμΦ and BMDMs. Alanine accumulation in both M2-stimulated cell types (Figure 6D) probably suggests that 2-OG can transaminate to Glu, which is a source through which Gln can replenish uridine diphosphate *N*-acetylglucosamine (UDP-GlcNAc), which is necessary to glycosylate M2 markers [28,29]. Arginine metabolism was enhanced following M1 and M2 stimulation, but was significantly reduced in the M1-stimulated BMDMs compared to the unstimulated cells (Figure 6A). However, in both M1-stimulated cell types, citrulline levels strongly increased, in line with the *Nos2* overexpression typical of the M1 polarization state (Figure 6C). By contrast, in response to M2 stimuli, both cell types upregulated *Arg1*, resulting in ornithine accumulation, a precursor of polyamines in both iμΦ and BMDMs (Figure 6B). Interestingly, both citrulline and ornithine accumulation was enhanced in the BMDMs compared to the iμΦ. 

**Figure A3:**
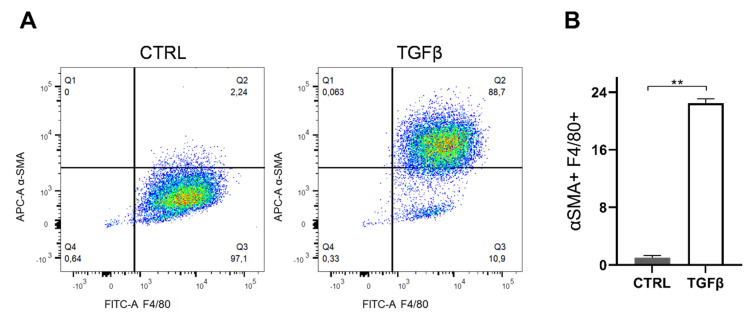
TGFβ stimulation on iμΦ leads to increase of αSMA. (**A**) Gating strategy for the expression of the macrophage marker anti-F4/80-FITC and the myofibroblast marker αSMA-APC by flow cytometry in iμΦ after a 5 day treatment with TGFβ (5 ng/mL). Unstimulated iµФ were used as a control (CTRL). (**B**) Quantification of the αSMA^+^ cell population (*n* = 3). Data are means ± SEM. ** *p* < 0.01.

Since in vitro primary BMDMs under TGFβ stimulation acquire fibrosis-inducing features, a process that in the kidney may occur through a macrophage-to-myofibroblast transition (MMT) [23,24], we tested the possibility that iμΦ might also be able to acquire these features. As reported in Figure A3, under TGFβ stimulation, the iμΦ displayed similarities with the primary corresponding cells, as the immortalized cells increased the levels α-SMA, which is a marker of profibrotic macrophages.

All these data clearly indicate that iμΦ stimulated toward the M1 or M2 state display the main phenotypical and metabolic features of BMDMs and can be therefore considered a suitable model to investigate the mechanisms mediating macrophage function.

## 4. Discussion

Macrophages are considered key players in the tumor microenvironment [30,31,32]. Macrophages not only contribute to all the steps of metastasis as major immune cell type within the tumor microenvironment (TME) [33], but also colonize the premetastatic niche with the aim of priming the environment to allow the survival of disseminated cancer cells [33]. In addition, macrophages are responsible for the acquisition of resistance to traditional chemotherapy [33]. At each step of tumor progression, cancer cells are exposed to macrophages, which in principle could recognize immunogenic cancer cells and hamper their proliferation. For these reasons, targeting tumor-associated macrophages (TAMs) is now considered a promising strategy against cancer. Murine models of cancer are usually used to test different TAM-targeting strategies, among which are macrophage depletion, limiting macrophage recruitment and rewiring macrophage phenotypes from a protumoral to an antitumoral function [34]. The discovery that the different polarization states of macrophages are underlined by specific metabolic features [27,35,36] has prompted scientists to evaluate the targeting of metabolism as a promising strategy to polarize TAMs toward a more antitumoral state without hampering viability, with the objective of reducing cancer growth and metastasis development [35]. Despite this need, our knowledge of the mechanisms related to the control of cancer and the metastatic cascade is still limited. Studies into mechanisms regulating innate immune activation often require the availability of genetically modified mice, not only for in vivo but also for in vitro studies, especially for the understanding of the underlying molecular mediators of in vivo effects. The fact that macrophages are generated from these mouse strains requires a high investment in terms of costs and spaces for animal housing and breeding. Immortalized macrophage cell lines might help to respond to this need. There is a general consensus that, when referring to in vitro polarization, the binary and static M1–M2 polarization model fails to describe the features of macrophage polarization in vivo, which occurs dynamically in a tissue-specific fashion. However, in vitro polarization studies might offer the opportunity to unravel novel checkpoints, obtaining molecular insights into the mechanisms underlying in vivo evidence and to evaluate, in a high throughput fashion, the functional significance of perturbing specific metabolic and signaling pathways. A reliable cell system that consistently responds to stimuli could therefore be used to investigate whether a drug, gene knock-down or other conditions influence macrophage polarization and function or determine the rewiring of an acquired polarization state. 

Here, we present an immortalized cell line obtained from BMDMs. The immortalization protocol can be easily repeated to obtain genetically modified immortalized macrophages, facilitating the investigation of relevant pathways of immune function and their underlying molecular mechanisms. A comparison with the primary counterparts clearly indicates that these cells not only mimic the phenotype but also display similar metabolic features (although to a different extent) to primary macrophages under LPS/IFNγ or IL-4 stimulation. The immortalization process does not affect the capacity of differentiation under LPS/IFNγ or IL-4 as, after seven days of differentiation, BMDMs and iµΦ do not display any difference in terms of their morphology or classical glycoproteins (CD38 for M1 and CD206 for M2) expression. Both cell types display the typical markers of M1 and M2 polarization states to a similar extent, although for M2 polarization only IL-4 stimulus was tested. Furthermore these cells exhibit the effects of proper inflammasome assembly following M1 stimulus, similarly respond to TGFβ, and display some of the main metabolic features associated with M1 and M2 polarization states. Classically activated iµΦ share with primary M1 macrophages the main features of M1 metabolic response, such as the reduction of oxygen consumption linked to the interruption of the TCA cycle with citrate and succinate accumulation. Succinate promotes HIF1α stability by inhibiting prolyl hydroxylases (PHDs), a class of 2-OG-dependent dioxygenases that regulate HIF1α expression in an oxygen-dependent manner, thus preventing HIF1α degradation in the presence of oxygen [37]. Indeed, both classically activated cell types display a lower 2-OG/succinate ratio. As a consequence of the downregulation of isocitrate dehydrogenase [25] and the upregulation of the mitochondrial citrate carrier CIC [38], citrate is exported into the cytosol, where it supports NO, ROS and fatty acid synthesis (FAS), which is a typical feature of M1 macrophages [27] that is necessary for prostaglandin E2 (PGE2) synthesis [39,40]. Furthermore, classically activated cells similarly reroute arginine metabolism toward citrulline and the synthesis of nitric oxide (NO), which contributes to the suppression of mitochondrial metabolism by inhibiting aconitase 2 and mitochondrial electron transport chain (ETC) complexes [41]. Alternatively polarized immortalized cells display features of the M2 metabolic response, in line with the corresponding primary cells. Indeed the 2-OG/succinate ratio is higher compared to the corresponding M1-stimulated cells, which is the typical metabolic feature of alternatively activated macrophages associated with the activation of PHDs and HIF1α destabilization. This finding is in line with the bioenergetic data from both cell types, which clearly indicate an active mitochondrial respiratory activity. This associates with the rewiring of arginine metabolism from NO to ornithine synthesis in both M2-polarized cells, which is the precursor of polyamines that are typically produced in M2-polarized cells to control tissue repair [42]. 

It is interesting to note that the % changes in the level of the metabolites followed a similar trend in both the M1- and M2-stimulated cell types, but the % variations were much higher in the BMDMs than in the iµΦ in the case of citrulline, ornithine and citrate. Indeed, the measured basal levels of citrulline were lower in the MΦ BMDMs compared to the iµΦ; this partially accounts for the higher % citrulline increase in the M1-stimulated BMDMs compared to the iµΦ. This difference is probably linked to the *Nos2* expression levels, which reached the same value in the M1-stimulated cells but were set to a much lower value in the unstimulated BMDMs compared to the iµΦ (see Figure 1). The subsequent higher citrulline production in the M1-BMDMs was accompanied by higher NO levels, which can inhibit aconitase to a higher extent in M1-stimulated BMDMs compared to M1 iµΦ, leading to a higher citrate accumulation in the M1-stimulated BMDMs compared to the iµΦ. The amount of NO released due to the higher *Nos2* expression levels in the unstimulated iµΦ probably did not exceed the threshold level necessary to elicit aconitase inhibition [41]. Furthermore, some differences could also be ascribed to the expression of *myc* by the iµФ cells, particularly v-*myc*, which is the viral homolog of c-*myc* [43,44] Indeed, the % decrease in the 2-OG/succinate ratio following M1 stimulation was higher in the BMDMs (50% decrease) than in the iµФ (35%). This can probably be ascribed to the higher glutaminolytic flux driven by *myc* in the iµΦ compared to the BMDMs [45], which resulted in slightly higher 2-OG levels in the immortalized M1-stimulated cells. Similarly, the lower ornithine % increase in the M2-stimulated iµФ compared to the BMDMs might be explained by the fact that ornithine decarboxylase (ODC) is strongly upregulated by *myc* [46], which would more extensively channel ornithine into putrescine synthesis.

## 5. Conclusions

Overall, our data indicate that the immortalized cell line obtained from BMDMs polarizes toward an M1 and M2 status, maintaining the typical features of the primary counterparts both from a phenotypical and a metabolic point of view, although with some differences in the metabolic % changes. This cell line could be used to obtain molecular insights into the mechanisms associated with macrophage rewiring and to unravel the functional significance of perturbing specific pathways.

## Figures and Tables

**Table 1:** Primers used for RT-PCR.

Target	Forward	Reverse
*Rplp0*	GGGCATCACCACGAAAATCTC	CTGCCGTTGTCAAACACCT
*Il1b*	GGACATGAGCACCTTCTTTTCC	TTGTTCATCTCGGAGCCTGTAG
*Nos2*	GCACATTTGGGAATGGAGACTG	GGCCAAACACAGCATACCTGA
*Tnf*	GAAAAGCAAGCAGCCAACCA	CGGATCATGCTTTCTGTGCTC
*Il6*	AGGATACCACTCCCAACAGAC	GCCATTGCACAACTCTTTTCTC
*Il12b*	AGACATGGAGTCATAGGCTCT	CCATTTTCCTTCTTGTGGAGC
*Cxcl10*	ATCATCCCTGCGAGCCTATCCT	GACCTTTTTTGGCTAAACGCTTTC
*Tgfb*	TATTGCTTCAGCTCCACAGAGA	CAGACAGAAGTTGGCATGGTAG
*Il10*	GGTTGCCAAGCCTTATCGGA	ACCTGCTCCACTGCCTTGCT
*Mrc1*	TTGCACTTTGAGGGAAGCGA	CCTTGCCTGATGCCAGGTTA
*Erg2*	CCTTTGACCAGATGAACGGAGTG	CTGGTTTCTAGGTGCAGAGATGG
*Arg1*	ACAAGACAGGGCTCCTTTCAG	GGCTTATGGTTACCCTCCCG
*Fizz1*	CCTGCTGGGATGACTGCTAC	CAGTGGTCCAGTCAACGAGT

## Data Availability

The data presented in this study are available on request from the corresponding author.

## Supplementary Materials

The following are available online at https://www.mdpi.com/article/10.3390/cancers13215478/s1, Figure S1: Original blot images of Figure 3A representing NOS2, ARG1, and actin (as reference protein) in resting (MΦ)-, M1- and M2-stimulated iμΦ or BMDM macrophages.

## Appendix

Figure A1 depicts a representative image of the FACS gating strategy to evaluate the M1- and M2-polarized macrophages from the immortalized and primary cells.

Figure A2 displays the images of resting (MΦ)-, M1- and M2-stimulated iμΦ and BMDM macrophages.

Figure A3 displays the effects of TGFβ stimulation on iμΦ.
