# Supplementary material for: The J2-Immortalized Murine Macrophage Cell Line Displays Phenotypical and Metabolic Features of Primary BMDMs in Their M1 and M2 Polarization State
Source: Cancers (Basel). 2021 Oct 31;13(21):5478. doi: 10.3390/cancers13215478 (PMC8582589; doi:10.3390/cancers13215478)
Supplement: Supplementary file 1 [file cancers-13-05478-s001.zip › cancers-1417697-supplementary.pdf]

Anti NOS2

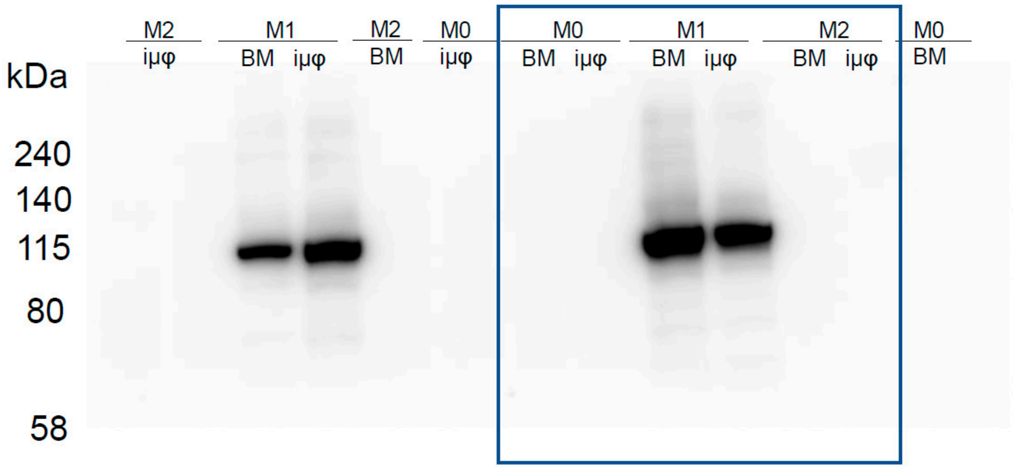

Anti ARG1

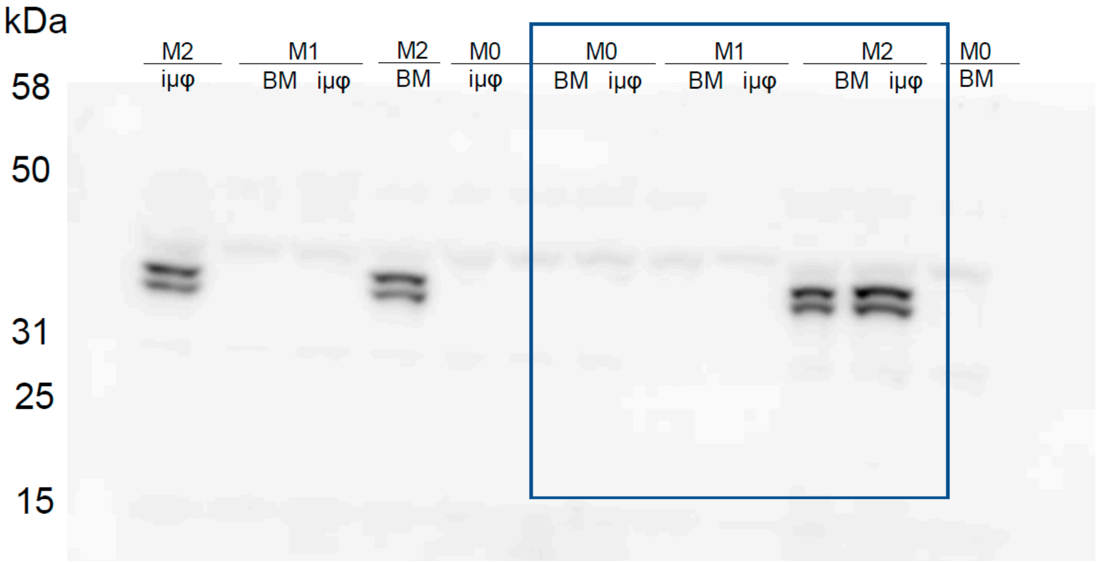

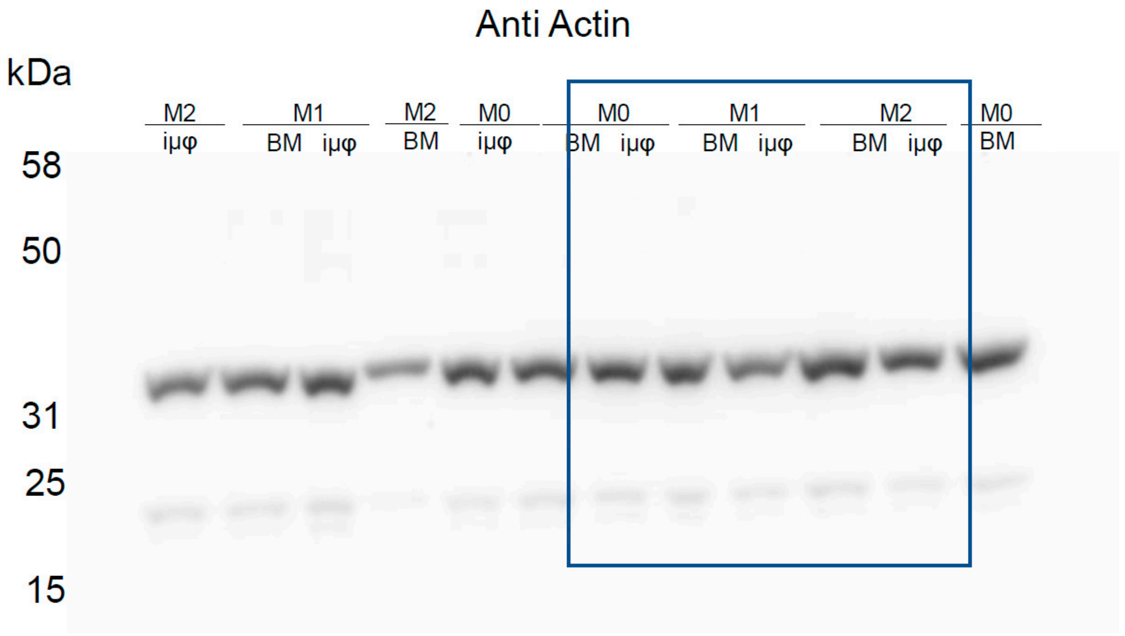

Figure S1. Original blot images of Figure 3A representing NOS2, ARG1, and actin (as reference protein) in resting (MΦ)-, M1- and M2-stimulated iμΦ or BMDM macrophages
